# Cancer in the head and neck region and its face under the perspective of users of a public reference hospital in Recife, state of Pernambuco, Brazil^☆^^☆☆^

**DOI:** 10.1016/j.bjorl.2025.101601

**Published:** 2025-06-04

**Authors:** Angélica Lopes Frade, Beatriz Santos Borges, Fátima Cristina Mendes Matos, Aurora Karla de Lacerda Vidal

**Affiliations:** Universidade de Pernambuco, Hospital Universitário Oswaldo Cruz, Centro de Oncologia (CEON/HUOC/UPE), Recife, PE, Brazil

**Keywords:** Head and neck malignant neoplasms, Humanization of health care, Qualitative research

## Abstract

**Objective:**

This study aimed to identify and analyze the difficulties experienced by head and neck (H&N) cancer patients, who are users of the Brazilian public health system (SUS), from their perspective of diagnosis to post-treatment.

**Methods:**

Qualitative case series carried out using individual semi- structured interviews. Data were collected from October 2019 to March 2020 and interpreted by content analysis.

**Results:**

Three categories emerged from the analysis: “difficulties in the diagnosis phase”, “conflicts experienced during treatment” and “post-treatment difficulties/sequelae”. Corroborating the literature, it was found that the difficulties faced by cancer patients are present in all stages of the disease: from access to prevention health services and diagnosis to post-treatment, influenced by late diagnosis, treatment side effects, and disease comorbidities.

**Conclusion:**

It is essential to carry out studies addressing changes in the family, professional and personal scope of cancer patients, aiming to provide them with comprehensive care and health professionals with understanding about what this disease represents in the life of these individuals.

**Level of evidence:**

Level IV.

## Highlights

• It was observed difficulties in the diagnosis phase.
• They experienced difficults on family support.
• Who to seek and how to deal with post-treatment difficulties/sequelae.

## Introduction

Cancer is a public healthproblem in the Brazilian public health system because of its epidemiological, social and economic amplitude. The incidence of cancer has grown worldwide as a result of changes in the population age profile, increased exposure to risk factors and/or improved diagnostic technologies. According to the Brazilian National Cancer Institute (INCA), there will be 625,000 new cases of cancer each year of the 2020–2022 three-year period, including 7,650 in the larynx, 11,390 in the esophagus, 13,780 in the thyroid, and 15,190 in the oral cavity.

Prevention is still the most effective method to combat Head and Neck (H&N) cancer, and is directly associated with avoidance of unhealthy habits, such as smoking and excessive alcohol intake, as well as of some sexual intercourses, especially oral sex without protection because of the possibility of HPV infection, which are risk factors for tumors in this region.^1^, ^2^, ^3^, ^4^

Unfortunately, H&N cancer has a high mortality rate in Brazil because most patients are treated in advanced stages of the disease, culminating in late diagnosis and, consequently, worse prognosis and smaller chances of survival. From this perspective, early diagnosis is the biggest challenge in this area to minimize the occurrence of post-treatment sequelae and favor a quicker return of patients, who see themselves as seriously ill, to social, personal and professional life, thus mitigating their conflicts in the psychosocial sphere.^5^

This progressive increase in the demand for diagnosis and treatment, as well as the need to ensure universality, equity, and completeness in the care for cancer patients, led the Ministry of Health (MS) to create the National Cancer Control Policy in its Health Care Network for People with Chronic Diseases within the scope of the Unified Health System (SUS), established by Ordinance nº 874 of 16 May 20136.

This national cancer care policy aims to reduce the mortality and disability caused by the disease, as well as to prevent and detect it early, and to treat and rehabilitate these patients or offer them palliative care. The objective is to reduce the incidence of some types of cancer and improve treatment conditions and survival rates for patients. These measures are an important ally to face the challenges that the progression of the disease’s incidence brings to Brazilian public health.^6^

Faced with the diagnosis of cancer, patients are always taken by feelings of pain, suffering and death, which permeate the entire disease process, from diagnosis to treatment, affecting the social, emotional, family and professional spheres of these individuals. Despite all the repercussions in the psychosocial context, there is a lack of qualitative studies addressing the conflicts experienced by patients, from their perspective, aimed at this more subjective and unique dimension. In contrast, most studies focus on the biological dimension of the human being, evidenced by so many advances in technology, therapy, and preventive measures in health.^1^, ^4^, ^7^

In this context, this study aimed to identify and analyze the difficulties experienced by H&N cancer patients, who are users of SUS, from their perspective of diagnosis to post-treatment.

## Methods

Qualitative research studies the subjective aspects of social phenomena and human behavior, considering the uniqueness of individuals and their subjectivity, analyzing their statements and interpreting their language.^8^, ^9^, ^10^, ^11^ Thus, a qualitative case series was carried out using individual semi-structured interviews, and documents – information contained in medical records – were analyzed.^12^

The sample was obtained by convenience observing the following inclusion criteria: patients aged ≥18-years, of both sexes, with a diagnosis of H&N cancer, who had received medical and dental oncological assistance, were under follow-up, and agreed to participate in the study by signing an Informed Consent Form (ICF). The data were collected at the Centro de Oncologia do Hospital Universitário Oswaldo Cruz da Universidade Estadual de Pernambuco (CEON/ HUOC/UPE), a reference center in the diagnosis and treatment of cancer located in Recife, state of Pernambuco, Brazil, from October 2019 to March 2020. This study respects the principles of autonomy and the norms for human research, and is part of a project approved by the Research Ethics Committee of the State University of Pernambuco under opinion nº 3.184.856.

The interviews were conducted in a private room while the patients were waiting for medical and/or dental assistance. To this end, a semi-structured questionnaire was applied individually. The questionnaire addressed the following phases of disease: diagnosis, understanding, acceptance and completion of treatment, and post-treatment, including resumption of personal, professional and social life and complications/sequelae. Participants were identified by the letter P and sequentially numbered (P1, P2, P3, etc.) according to the order in which the interviews were carried out, thus ensuring their anonymity. The interviews were terminated by data saturation.

The interviews were recorded using the application Smart Voice Recorder 1.11.0 (SmartMOB®) and then transcribed in full by the researcher. The information was analyzed using Bardin’s content analysis, which consists of three stages: pre-analysis, material exploration, and treatment of the results obtained.^13^ Three categories emerged from the analysis: “difficulties in the diagnosis phase”, “conflicts experienced during treatment”, and “post-treatment difficulties/sequelae”.

## Results

The study sample was composed of five male patients aged 39 to 67 years ‒ three of them were former smokers. Regarding marital status, four of the participants were married and one was a widower. Concerning level of education, one of them had completed High School and had a technical degree, one did not complete High School, and the other three did not complete Elementary School. As for antineoplastic treatment, three participants were submitted to chemoradiotherapy and the remaining underwent surgery and chemoradiotherapy, and surgery and radioiodine therapy.

Regarding diagnosis at admission to the CEON/HUOC/UPE, the participants presented oropharyngeal cancer, oral cancer in the retromolar region, and cancer in the glands, one in the submandibular gland and one in the thyroid. The last two participants presented metastasis according to information obtained during the interviews and confirmed by their medical records: distant and regional metastasis, respectively.

The collected data were grouped into three categories depending on the difficulties and conflicts experienced and reported: a) Difficulties in the diagnosis phase, b) Conflicts experienced during treatment, and c) Post- treatment difficulties/sequelae, as described below.

### Difficulties in the diagnosis phase

For the participants of this study, the cancer journey began with signs and symptoms that led them to suspect that something was wrong, and thus seek medical and/or dental assistance.

I had an appointment at a health center close to home to see my tonsils, which hurt, and the doctor pressed them and noticed that I had a lump on my neck, and asked me if it hurt, and I said it didn’t. Then he asked to open my mouth and saw this lump behind my tonsil (P2).

It started with some pain on the right side, then I felt a little lump on my jaw, and I looked for a dentist because I thought it was a tooth problem, but they said it wasn’t (P4).

There was a little lump on my neck, the size of a bean, and it got bigger, I didn’t feel any pain, I didn’t feel anything. I played soccer, drank some beers. But this little bump grew and, at election time, a candidate to office passed by with a doctor who worked at the outpatient clinic, then I said that I could vote, but that I needed someone to look at this painless lump that was growing suddenly. Then he asked the doctor to examine me (P5).

Difficulty in accessing treatment contributes to late diagnosis, as it can be observed in the reports below: […] *my sister was hiding it from me, but I already knew because I’d had private exam that my sister and my niece had paid to advance the treatment* (P5); […] *there was a delay in getting the result after the surgery because they sent it to Fiocruz and it took a while to return* (P3).

However, after being correctly referred to the hospital where they would undergo their treatment, the participants reported that they had access to consultations, exams, surgical procedures, chemotherapy, and radiotherapy without difficulties. Therefore, it is verified that they had access to health services, as explained in the statements below. However, the quality of these services can be questioned, both because of the difficulty in the initial access to them and the delay in starting treatment and even having exams; one of the participants had to use his own financial resources to have access to them, as seen in the statement above (P5).

There was no delay in treatment. It was discovered right away; I was operated immediately (P1).

Everything went smoothly for me to come and have the treatment here, and I am still being followed up with exams (P2).

I had no difficulty in carrying out the treatment here at the hospital, I had chemotherapy and radiotherapy without problems (P4).

The emotional impact experienced by patients can be observed in the following statements, when they were asked about the diagnosis phase:

*When I found out, I didn’t feel well at all. I kept thinking, this disease kills even rich people, let alone a poor person like me! It wasn’t easy at all* (P2).

[…] *when they say “it’s cancer”, the first thing that comes to mind is “I’m going to die.” I got nervous, psychologically my world collapsed* (P1).

*I faced it naturally* (P4).

*I accepted everything in a good way, because I really do* (P5).

*When they told me it was cancer, I was calm. It would be worse if I despaired. I accepted my situation and went after treatment right away* (P3).

*My family and I did not have the clinical view of a doctor – that there are more than 250 types of cancer, that some more others less aggressive, that there are skin cancers – and I was advised that thyroid tutor, although malignant, is a type of cancer that has a 99% cure rate ‒ a very good prognosis. So when I heard that, psychologically, I felt good, you know* (P3).

*I thought I was going to die! Because we don’t understand the whole clinical process, don’t have this view of the disease, are lay people who are not trained for this. So we get upset, that’s the reality* (P2).

*But thank God above all, as well as medicine, which God created to help with the treatment and the things we have to do, with the guidance of some doctors, we have this maturity in the treatment and learn to deal with the disease* (P1).

*And the doctor also told me that people who drink alcohol and smoke have a 50% greater chance of having cancer. There was no point in visiting the doctor if I didn’t give up these habits. Today I’m strong here because I’ve been following the doctors’ and dentists’ advice* (P5).

*One of the physicians told me, “my brother died of this”. I even cried. How can a doctor say that to me? Then I asked my sister, who worked here, to find another doctor, because he shouldn’t have told me that. But I didn’t say anything bad to him because my sister worked here, and because I don’t like to criticize anyone. But I didn’t want to be attended by him anymore* (P4).

### Conflicts experienced during treatment

The difficulties linked to cancer experienced by the participants and the importance of family support are exemplified in the following statements:

*Family support is a blessing, because there are families that don’t provide it, but I have a family that looks after me, that likes me* (P3).

*I relied on my family, when I needed, I asked them for help. When I was in this difficult situation, everyone helped me a lot, thank God* (P1).

*We don’t have that look to say, “I have a disorder”. I say, “I’m not fine”, and when we’re going through that, we don’t realize it, who usually notices it is the person on our side, in my case, it was my wife. She received all this impact and gave me strength. She thought, “My husband is going crazy, he’s depressed”* (P4).

*My brother-in-law took me to every radiotherapy session* (P5).

Another important finding concerns the difficulty in acquiring medication during treatment and its financial implications. As noted in the statement below:

*I went through financial difficulties because I had to stop working and I received very little. Where I used to work, at the gambling stall, they paid as they please, and I asked a lot there, for them to send me medicine. I would go there with the prescription and they would send the medicine* (P4).

Another difficulty experienced and reported by the participants concerned the side effects resulting from chemotherapy and radiotherapy. As evidenced in the following statements:

*I started using a tube to eat, I was reduced to skin and bones, you can’t imagine. The food went in the wrong place because the tube was dislocated, so I went to an emergency room and I almost died, I was cold as a stone* (P5).

*I felt a lot of pain after the chemotherapy*. (P4).

[…] *I was already undergoing radiotherapy, but it took long to be assisted there, there were people who gave me their turn because I was in a wheelchair, I was so weak* (P5).

*And you get a little retarded because of your hormones are working all out of control, and since I don’t have these hormones because my thyroid was removed, I need medication to control this situation. But it’s not the same, I don’t feel like it’s the same thing. I feel there’s something different because of the effect of the hormones* (P3).

[…] *and the other radiation was so high that I had to spend three days in hospital until it lowered, and they told me to drink a lot of water, take a shower in the hospital* […] (P3).

*I often have memory loss as well as muscle weakness, once I was so weak that I spent a week lying in bed and couldn’t get up* (P1).

*I was terrified at home, when I felt a lot of pain, so I took medicine because the pain was too intense, so I was like crazy at home. There were people who commented that I wouldn’t last even a month* (P2).

*They canceled this radiation session, which would be the third, because I complained about mouth dryness and that it would become even worse, so they told me to take a break and followed me up* (P1).

About living with the disease, the participants expressed a series of thoughts and reflections on life, as reported below:

*I started giving less value to the things we can achieve, to try to help people more. Seeing people differently, looking at the world with a panoramic view, that is, having a new chance to do everything differently* (P1).

*Today I have a purpose in my life that I didn’t have before, I had a goal in life: study, graduate, earn money, support my family, educate, and that was it. Now I have a completely different view* […] (P2).

*I stopped selling my beer, because God has done so much good to me that I will no longer sell beer and listen to worldly music* (P4).

In addition to changes in the singular and more subjective aspects of the individual’s psychological and spiritual spheres, adjustments to everyday life were also reported, including strict monitoring of their health status, as exemplified in the following statements:

*I was submitted to total thyroidectomy in 2011, of both thyroid lobes, right and left, then I underwent radioiodine therapy and had a recurrence in 2014* (P1).

[…] *Three more nodules appeared when I did the aspiration puncture to detect this abnormality, then it showed again, and with metastasis, it invaded the lymph nodes of the neck, so I had to undergo a cervical resection of this affected area and a new radiation therapy; this was in 2014. Since then, I’ve been under follow-up with exams and taking medication* (P1).

[…] *I’ve been under follow-up to this day because of my lung. In all, I’ve had three surgeries for free here at Oswaldo Cruz, Jesus is the one who pays for these doctors’ blessings, including the dentists’, who are also still following me up.* (P4).

*But I’m still under follow-up, right, being treated. And what I’m up to now here at Oswaldo Cruz is the prostate treatment* (P2).

*I only do the exams every six months because the disease may come back. So I’ll have to be monitored for the rest of my life* […] (P3).

There are also tensions generated by possible complications arising from the treatment or the disease itself, which often require the signature of terms of responsibility given the risks of the surgical procedures, as noted below:

*In fact, my wife and my two daughters signed a term, because on this side here is where he said that all innervation passes, veins that go to the heart, and that could even lead to death. And in case something went wrong with that nerve, I wouldn’t be able to move it, I’d have a saggy and drooping face* (P2).

The statements below expose the pain, depression, and risk of suicide in these patients:

*I got depressed, I stayed inside a locked room, isolated, sad. That was all I thought about, I thought I was going to die* (P1).

*You feel your heart beating, you can’t sleep well, your heart starts beating fast, you feel very anxious and short of breath, a lot of cold sweats, dizziness, headache, and as you can’t sleep well, you feel sleepy all day long* (P2).

*I thought about suicide twice, not because I wanted to, you know, but that’s what was on my mind* (P1).

*I have this self-remorse, because until today my head has been a bit disturbed, it’s not the same anymore. There are times when I’m happy, there are times when I get angry, I notice that I’m talking too fast, in agony, talking nonsense. To this day, I still take prescription medication, because this disease has really messed my head up* (P3).

Concerning the search for psychological and psychiatric assistance, it is possible to identify stigmatization, which is still very strong with regard to mental illness, as observed in the following statement:

“[…] Having appointments with psychologists and psychiatrists, who prescribe medicine to simply control the nerves. People say these medicines are for mad people, but they’re not.” (P1)

Analysis of the statements showed that most of the interviewees used alternative/complementary remedies, and the most used was individual prayer, thus demonstrating the belief in the effectiveness of prayer in their healing.

*Then I said, “It’s not you who’s going to do this surgery, it’s Jesus who’s going to do it. The Lord will follow through on what He tells you to do* (P4).

*I go to church every Sunday. I don’t know if I’m a believer, only Jesus does, because there are so many people who say they are believers but don’t follow in Jesus’s footsteps. I’ve also stopped selling my beer, because God has done so much good to me that I will no longer sell beer* (P3).

*I wasn’t a believer at the time I found out I had cancer, but I knelt at home listening to gospel radio and asking God for help every night* (P2).

*I began to get closer to God, because I am a Christian, an evangelical Christian* (P1).

### Post-treatment difficulties/sequelae

Regarding post-treatment difficulties/sequelae, in addition to what was included in the medical records, some of the symptoms described were present in the interviews:

*To eat I have to drink water to help swallow […] I started feeling this dryness in my mouth, very dry mouth, complete absence of saliva* (P1).

[…] *the throat keeps closing because of lack of saliva, I can’t swallow properly (P3).*

*The only things that remained were the scar, the stiff neck, and a very dry mouth. But what really bothers me is the dry mouth, the lack of saliva* (P5).

*The only sequelae I had were this scar, the stiff neck, and this problem with my teeth, which I can no longer pull out* (P2).

[…] *I feel a lot of pain because of this bone wound and I can’t chew properly, I can’t even chew things like smoothies and juice anymore. I have to eat using a tube* (P4).

The following statements show what patients think about the health professionals, commitment, and health care for the population, as well as how they acknowledge all the oncological care provided.

Because you are here to fulfill a mission, not only because of your study, but because of that commitment to be a doctor, and God is guiding you. You are one of the people who are saving our lives, because God gave you experience. God has sent you to take care of His children, like myself and many others out there (P2).

I thank everyone who works here at the hospital, because everyone has been very good to me. When I come here for appointments, I always talk to and hug the doctors who attended to me, because I recognize what they’ve done for me (P5).

Thus, the results show that the difficulties faced by cancer patients were present in all phases of the disease, from access to health services, diagnosis, to the post-treatment phase, with the influence of late diagnosis, side effects of treatments, and disease comorbidities.

## Discussion

Zanelli^14^ pointed out that it is important to verify whether the content of a verbalization corresponds to a documentary source, as it was done in the present study.

Depending on the difficulties and conflicts experienced, reported and analyzed, specifically on the difficulties in the diagnosis phase, P5 negotiated his health, with medical assistance in exchange for voting, which is still a quite common practice in Brazil. However, Art. 2 of Law nº 8080 of 19 September 1990 states that “Health is a fundamental human right, and the State must provide the indispensable conditions for its full exercise”.^15^ It is thus observed that, although access to health is a guaranteed right of Brazilian citizens, in practice, the assistance offered presents a series of obstacles, making it constantly questioned.

It is also noted that the patients only sought assistance after the appearance of “lumps”. Perhaps, culturally, the symbolism of masculinity in society is linked to the meaning of invulnerability, strength, courage, and virility. Therefore, the disease process could compromise their masculinity, thus making them delay the search for medical assistance as much as possible. Thus, it is essential to adopt measures that address this cultural prejudice to minimize late diagnoses and, consequently, increase the chances of survival.^16^

Another factor for late diagnosis is associated with the difficulty in accessing health services. Despite the struggle for early diagnosis of cancer through the development of social and economic policies aimed at facilitating access to health actions and services, in practice, there are still many barriers to it, often due to bureaucratic issues in the health care network and to demand much greater than supply,^17^ as observed in the statements by P5 and P3.

With regard to cancer, early diagnosis with immediate initiation of treatment is directly related to increased chances of survival. Therefore, it is essential that the health care network have all the necessary devices, such as clinical, surgical and laboratory specialties and trained professionals to maintain patient follow-up, as well as support and diagnostic methods for resoluteness in patient care oncology. Hence, delays in diagnosis and, consequently, in therapy contribute to disease dissemination and tumor growth, resulting in poor prognoses and lower chances of survival.^18^

It is expected that the news of a cancer diagnosis can have an important emotional impact, arousing feelings such as anger, anguish, impotence, helplessness, sadness, fragility, restlessness, anger and, especially, fear of death. Also, cancer carries with it the stigma of a fatal disease because, historically, the mortality rates related to it are considerably high despite all the scientific advances in the diagnosis and treatment of malignant neoplasms,^19^ as observed in the statements by P1 and P2.

In addition, P2 pointed to an association between health and purchasing power, that is, those with high socioeconomic statuses have greater access to medical assistance and cutting-edge treatments. And this is one of the reasons why equity is a principle of SUS, which aims to reduce inequality. Although cancer still carries with it the stigma of a fatal disease, some interviewees showed acceptance and resilience at the time of diagnosis, seeing it as just another obstacle in life, as evidenced in the statements by P4 and P5.

Therefore, it is essential that health professionals have qualified listening to meet the individual needs of cancer patients. This means clarifying all patients’ doubts and providing them with guidance and information about the disease, aiming to demystify it, thus facilitating patient adherence to the adopted therapy, which is crucial in the journey towards healing, as well as in relieving the tensions generated after disease diagnosis, as expressed by the interviewees.

However, when there is a lack of preparation by the health professional regarding the conduct of treatment in a way that aims to reassure the patient, it can culminate in the worsening of the individual’s emotional state and of the stigma attached to cancer, as observed in the statement by P4.

Considering the conflicts experienced during treatment, it was possible to identify the difficulties linked to cancer experienced by the interviewees that lead to a state of suffering, as well as the need for financial support, since part of the family budget is allocated to the medications necessary for treatment. The approach between patients and their families during the disease process assists them in going through this process with more resilience and perseverance, facilitating adherence to treatment and consolidating family ties. Thus providing a sense of security and strength in a moment of fragility.^20^ These findings are present in the statements of the participants in this study.

Another important finding expressed by the interviewees was the difficulty in accessing medications during treatment, often requiring the use of their own resources to acquire them. Thus, we note that health care is not being provided in full, contrary to what the Federal Constitution states. If patients have financial difficulties during oncological treatment, they must have their individualized and free treatment guaranteed, from exams to the prescription of medications, such as opioid analgesics and others, for the control of nausea and all other comorbidities caused by the antineoplastic treatment.^21^

Another difficulty experienced and reported by the patients concerned the side effects of chemotherapy and radiotherapy during treatment, inflicting great physical suffering, such as pain, nausea, vomiting, weight loss, fatigue, difficulties in eating due to loss of taste or even oral mucositis, and reduced salivary flow, resulting in this case from radiotherapy in the H&N region. This physical suffering contributes to emotional and psychic fragility because of the difficulty in living with the disease, and even with the aggression of the treatment. As shown in the interviewees’ statements.

These side effects can have a great influence on treatment withdrawal because they cause much physical suffering, leading to decreased quality of life, as they affect sleep, mood, diet, and routine in general. The decrease or loss of quality of life is directly linked to reduced treatment adherence, which may even culminate in the abandonment of antineoplastic therapy or the impossibility of continuing it, as reported by P1.

As observed in the reports of this study, living with the disease brings a series of thoughts and reflections about life, leading individuals affected by cancer to re-signify many aspects because of the new condition imposed, when they and their families start to live with a serious disease that results in changes in their personal and professional plans, thus compelling people to seek a purpose or meaning to their existence.^17^

The respondents reported changes in unique, subjective aspects of their psychological, spiritual and everyday lives; after all, there are chances of disease recurrence and, if it occurs, it is preferable that the diagnosis be made as early as possible so that antineoplastic treatment can be started, and a better prognosis is foreseen. Thus, they began a lifestyle marked by concerns and tensions generated by the rigorous and periodic practice of carrying out tests for early detection, in cases of disease recurrence, as well as by fear due to possible complications arising from the treatment or the disease itself.

Depression is a quite common finding in cancer patients. It is a comorbidity in approximately 25% of them and affects adherence to antineoplastic therapy due, in part, to reduction in self-care caused by the disease.^22^ This occurs because the impact of cancer diagnosis and the changes caused by it, both physical and psychological, make the patient more predisposed to develop psychiatric disorders, mainly depression and anxiety. These psychological illnesses may be related to the side effects of cancer treatment, such as hair loss, vomiting, nausea, dry mouth, fatigue, mutilations, weight loss, among other physical changes, which may be temporary or permanent, thus contributing to the onset of depression. Pain and depression are associated with risk of suicide in cancer patients,^23^ as detected in some of the participants’ statements. The stigma that characterizes mental illness contributes to hindering the search of psychological and psychiatric assistance, as explained by P1.

In this context, the need for a multidisciplinary team with qualified listening and effective communication becomes evident, thus creating a good bond between the team and the patient that could detect depressive symptoms early, because cancer patients tend not to talk about their depressive symptoms, perhaps because they believe that appearing strong keeps the physician interested in curing them. On the other hand, oncologists tend not to question the patients, assuming that they will speak spontaneously if there are any symptoms of depression. Furthermore, the fact that both diseases (depression and cancer) have symptoms in common, such as depressed mood, discouragement, fatigue and weight loss, makes the diagnosis even more difficult. Moreover, there may be underestimation of symptoms by the health team, as they consider that a depressive mood is a normal characteristic in cancer patients.^22^

A qualitative study carried out by Siqueira^24^ in 2006 revealed that cancer patients, when faced with the need to live with a serious health problem, seek alternative/complementary methods to cope with the disease, which differ from those offered by technical/scientific medicine, among which practices based on popular knowledge and religiosity stand out, corroborating the findings of this study. As Carl Sagan^25^ said in 1995: “The notion that spirituality and science are somehow mutually exclusive does a disservice to both”.

As for post-treatment difficulties/sequelae, the patients’ reports in this study corroborate the literature. Studies have shown that oncological treatment may cause acute and/or late, transient and/or permanent oral and maxillofacial complications, such as partial or total loss of structures and buco-sinusal communication resulting from surgical treatments in the H&N region; as well as mucositis, xerostomia, hyposalivation, odynophagia, dysphagia and dysgeusia, in addition to fibrosis, dysphonia, trismus, radiodermatitis and osteoradionecrosis, especially in cases of radiotherapy performed in the H&N region, as radiotherapy in this region presents very intense side effects because of major alterations in the oral cavity, which may culminate in the use of a feeding tube or even treatment interruption, depending mainly on cancer staging (TNM), which will guide the choice of treatment.^26^

Prior oral adaptation is not always conducted because of the advanced level of the disease at the time of diagnosis, requiring the immediate initiation of antineoplastic treatment, or even because of the team’s lack of knowledge about the importance of this adaptation and/or of obstacles to the access to dental services that provide targeted assistance to cancer patients, thus increasing the risk of post-treatment complications such as osteoradionecrosis.

Therefore, oral care and follow-up by a dental team are needed at all stages of cancer treatment, aiming at adapting the oral environment to avoid or mitigate possible complications and/or sequelae that may arise during or after surgical treatment and/or chemotherapy and/or radiotherapy, especially when performed to treat H&N neoplasms, as these patients present significant morbidities, contributing to loss of quality of life, which is crucial for better adherence to the proposed treatment.^27^, ^28^

It is essential to carry out studies addressing changes in the family, professional and personal scope of cancer patients, aiming to provide them with comprehensive care and health professionals with understanding about what this disease represents in the life of these individuals.

## Conclusion

In health care practice, understanding is linked to intervention and, in this study, through the analysis of the patients’ statements, it was possible to broaden this vision and understand the individuals in their integrity and context.

As noted, the conflicts experienced by cancer patients are numerous, ranging from access to medical care, side effects, to sequelae, which bring much physical, psychological and emotional suffering and change the life context of these individuals. Thus, there is clear need for a multidisciplinary team capable of providing humanized and comprehensive care.

Measures that enable the functioning of the National Cancer Control Policy, which ensures the rights of cancer patients to diagnosis, treatment, rehabilitation, and palliative care, must be urgently prepared.

## Declaration of competing interest

The authors declare no conflicts of interest.
